# Integrative Bioinformatics Identification of Baicalein as a Phytochemical Inhibitor of CHEK1 in Serous Ovarian Cancer: A Multi-Stage In Silico Drug Discovery Approach

**DOI:** 10.3390/ph19081234

**Published:** 2026-08-05

**Authors:** Poojhashri Jayagopal, Sandhiya Prabhakar, Abhinand Ponneri Adithavarman, Somdatta Yashwant Chaudhari

**Affiliations:** 1 Department of Bioinformatics, Faculty of Engineering and Technology, Sri Ramachandra Institute of Higher Education and Research, Chennai 600005, India; poojhashrij@gmail.com (P.J.); sandhiyaprabhakarp@gmail.com (S.P.); 2 Department of Pharmaceutical Chemistry, Progressive Education Society’s Modern College of Pharmacy, Yamunanagar, Sector 21, Nigdi, Pune 411044, India; somu.chaudhari@gmail.com

**Keywords:** serous ovarian cancer, CHEK1, differentially expressed genes, molecular docking, molecular dynamics simulation, baicalein, prexasertib, ADMET analysis, bioinformatics, phytochemicals

## Abstract

**Background**: Serous ovarian cancer (SOC) is the most aggressive subtype of epithelial ovarian cancer, frequently diagnosed at advanced stages with poor prognosis and chemotherapy resistance. Checkpoint kinase 1 (CHEK1), a key regulator of the DNA damage response, is overexpressed in ovarian cancer, making it a promising therapeutic target. No study has systematically evaluated natural compounds as CHEK1 inhibitors in SOC through an integrative transcriptomic and computational framework. **Methods**: A meta-analysis of three GEO datasets (GSE27651, GSE36668, GSE54388; n = 83) was performed using ImaGEO. DEGs were identified at |log_2_FC| ≥ 2 and FDR < 0.05. Pathway enrichment, PPI network analysis, virtual screening of 40 phytochemicals against CHEK1 (PDB: 9CE4), 100 ns MD simulations, and ADMET profiling were conducted using established bioinformatics and computational tools. **Results**: A total of 511 DEGs were identified, with significant dysregulation of apoptosis, DNA repair, and cell cycle pathways. CHEK1 emerged as the central hub gene. Baicalein exhibited the highest binding affinity (−9.334 kcal/mol), surpassing Prexasertib (−7.2 kcal/mol). MD simulations confirmed complex stability, and ADMET profiling demonstrated favorable drug-likeness with zero Lipinski violations. **Conclusions**: CHEK1 is established as a validated therapeutic target in SOC, and Baicalein is identified as a computationally superior natural lead compound, warranting experimental validation in ovarian cancer models.

## 1. Introduction

Ovarian cancer continues to pose one of the most formidable clinical challenges in gynecological oncology. Among its histological subtypes, serous ovarian cancer (SOC) is by far the most prevalent and the most lethal, responsible for 70–80% of all epithelial ovarian cancer diagnoses worldwide [1]. The burden of this disease is not evenly distributed. In India, ovarian cancer ranks third among gynecological malignancies, and available registry data indicate that age-standardized incidence has climbed from 3.8 to 4.61 per 100,000 women between 2017 and 2021: a trend that reflects both genuine increases in disease frequency and improved ascertainment [1].

What makes SOC particularly difficult to manage is not solely its biological aggressiveness, but the circumstances under which it is typically diagnosed. The disease produces no early specific symptoms. Patients often attribute abdominal bloating, pelvic discomfort, or altered bowel habits to more benign conditions, and there is currently no validated screening tool that is capable of detecting SOC at a clinically meaningful early stage. The result is that roughly 70–80% of patients present with stage III or IV disease, at which point the tumor has spread beyond the pelvis and curative resection is rarely achievable [2]. Despite decades of incremental refinement in surgical technique and systemic therapy, five-year survival rates remain stagnant at approximately 49% [3]. This figure has changed little, and it reflects a fundamental limitation: current treatment strategies address the consequences of advanced disease, rather than its molecular drivers.

Understanding why SOC behaves as it does requires looking at its genomic architecture. High-grade SOC (HGSOC) is defined by near-universal TP53 mutation, present in over 96% of tumors, alongside pervasive genomic instability and widespread defects in homologous recombination [4]. These are not incidental findings; they are the mechanistic basis of the disease’s behavior. Disrupted DNA damage response signaling, impaired cell cycle checkpoint control, and compromised replication fidelity together create a cellular environment in which unrestrained proliferation is not merely permitted but actively sustained [5,6]. Critically, these same genomic features that drive tumors’ progression also underpin resistance to platinum-based chemotherapy, the backbone of first-line treatment in SOC [7]. When the machinery that should register and respond to DNA damage is already dysfunctional, drugs that work by inducing that damage lose much of their therapeutic logic.

Conventional anticancer therapies, including targeted small-molecule inhibitors, are frequently limited by systemic toxicity, poor target selectivity, and the progressive emergence of drug resistance, collectively necessitating the development of safer and more effective therapeutic strategies [8]. In this context, phytochemicals have emerged as a promising class of bioactive compounds, offering structural diversity, multi-target mechanisms of action, and comparatively favorable safety profiles [9]. These plant-derived compounds are known to modulate key oncogenic pathways, including apoptosis induction, cell cycle regulation, and signal transduction networks, thereby exerting broad-spectrum anticancer effects with reduced off-target liability [10].

Despite these advantages, the systematic identification of therapeutically relevant phytochemicals remains challenging due to the vast chemical diversity of natural products and the inherent limitations of traditional experimental screening approaches, which are frequently time-consuming, resource-intensive, and inefficient at scale [8]. Although recent advances in computational biology and artificial intelligence have enabled high-throughput virtual screening and multi-omics data integration, the existing studies remain largely fragmented, focusing either on transcriptomic profiling or compound screening in isolation, but rarely combining both within a unified analytical framework. Integrative pipelines that simultaneously leverage transcriptomic analysis, network-based target identification, and systematic phytochemical screening are therefore essential to improve the mechanistic depth and translational relevance of computational drug discovery in oncology [11].

The present study was designed to address this challenge through a rigorous, multi-stage computational pipeline integrating meta-analysis, network biology, and in silico drug discovery approaches. Publicly available microarray datasets of serous ovarian cancer were systematically analyzed to identify robust differentially expressed genes (DEGs) associated with disease progression. These DEGs were further explored through protein–protein interaction (PPI) network construction and topological analysis to identify key hub genes with potential therapeutic relevance. In parallel, a curated library of phytochemicals with reported anticancer activity was compiled and evaluated for drug-likeness, ADME properties, and toxicity, using computational screening methods. Finally, selected compounds were subjected to molecular docking and molecular dynamics (MD) simulations against prioritized target proteins to assess their binding affinity, interaction stability, and overall drug potential, thereby providing a comprehensive framework for the identification of novel therapeutic candidates against serous ovarian cancer.

## 2. Results

### 2.1. Meta Analysis and Identification of Differently Expressed Genes

Meta-analysis of three GEO datasets (GSE27651, GSE36668, GSE54388; 83 samples: 16 controls, 67 SOC cases) using ImaGEO identified 511 high-confidence DEGs: 298 upregulated and 213 downregulated in SOC, relative to controls (|log_2_FC| ≥ 2; FDR-adjusted *p*-value < 0.05).

### 2.2. Pathway Enrichment Analysis and Literature Validation

**Figure 1:**
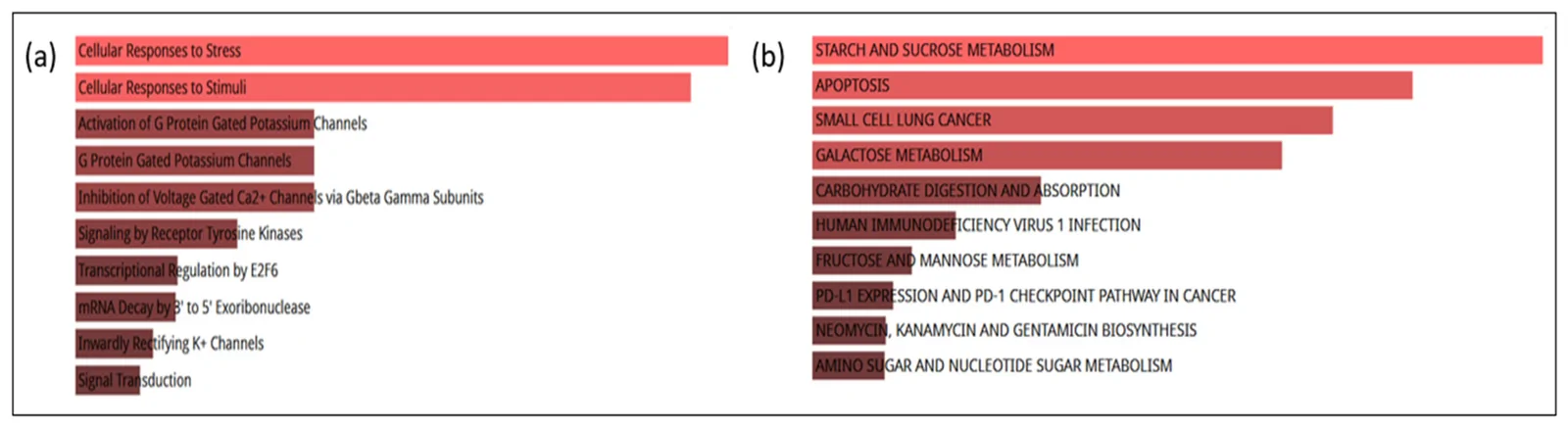
Pathway enrichment of differentially expressed genes in serous ovarian cancer. (**a**) Reactome 2024 pathways ranked by significance; eight validated by PubMed mining. (**b**) KEGG 2026 pathways showing shared cancer mechanisms. Darker red indicates higher significance.

Reactome-based analysis of 511 DEGs identified eight significant enriched pathways that are critical to SOC pathogenesis: apoptosis, DNA repair, cell cycle, disease, autophagy, DNA replication, programmed cell death, and metabolism (Figure 1a). Reactome was prioritized over KEGG as the primary database due to its more granular, human-specific pathway annotations [12,13]. Supplementary KEGG 2026 enriched identified additional enriched pathways including apoptosis, PD-L1 expression and PD-1 checkpoint pathway in cancer [14], and carbohydrate metabolism pathways (Figure 1b), complementing the Reactome results.

#### Literature-Based Pathway Validation

PubMed co-occurrence mining independently validated all eight enriched pathways as statistically significantly associated with SOC (Table 1). DNA repair (OR = 31.89; adjusted *p* = 2.59 × 10^−17^) and apoptosis (OR = 10.58; adjusted *p* = 1.81 × 10^−27^) demonstrated the strongest bibliometric associations.

### 2.3. PPI Network Construction and Hub Gene Identification

**Figure 2:**
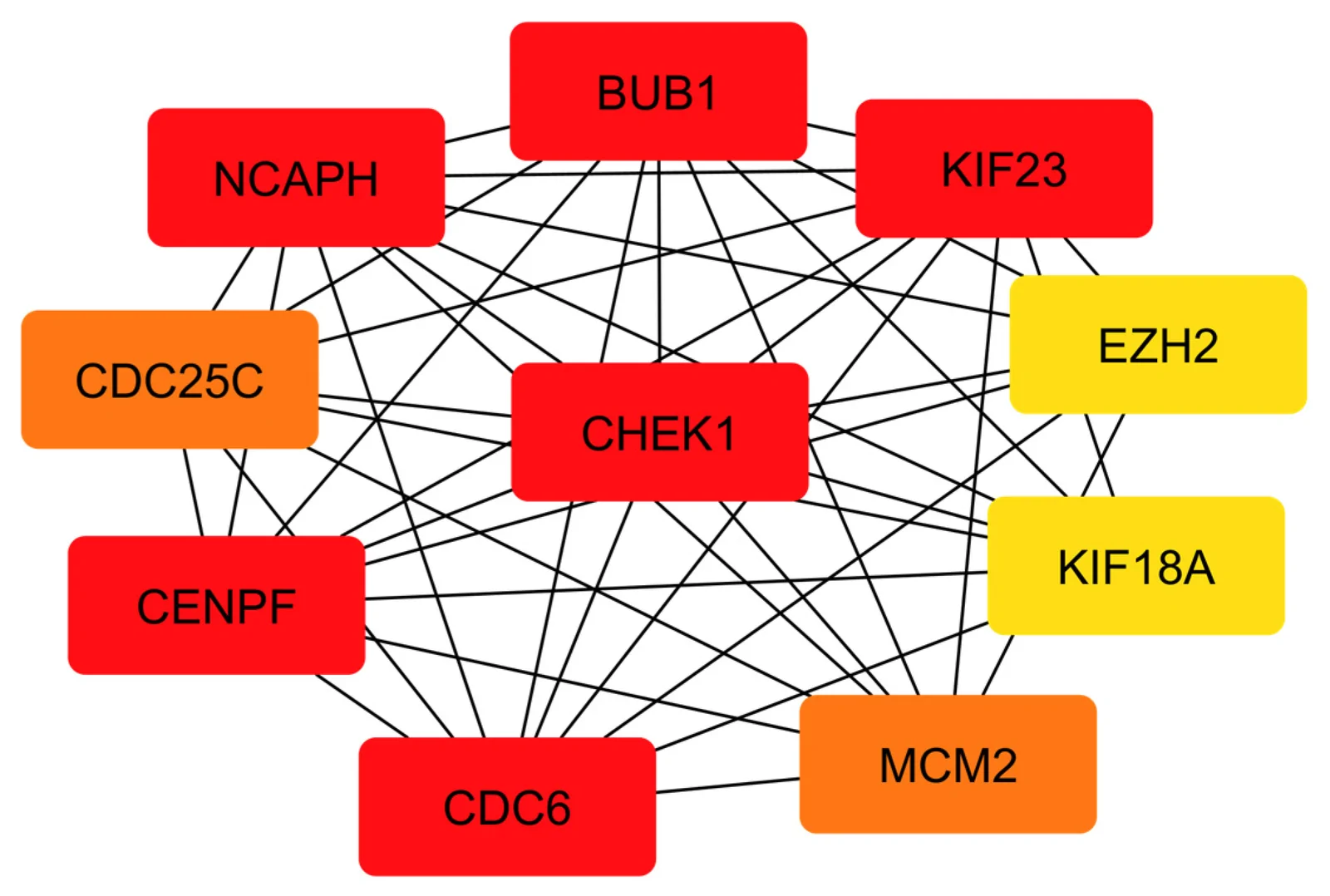
Protein–protein interaction (PPI) network of hub genes. Nodes represent proteins and edges indicate interactions. Colors denote connectivity: red (high), orange (moderate), and yellow (low).

A total of 109 unique genes from the eight validated pathways were submitted to STRING for PPI network construction. The network demonstrated dense connectivity among genes involved in cell cycle regulation, apoptosis, and DNA repair. Cytoscape-based MCODE and CytoHubba analysis identified CHEK1 as the central hub gene with the highest network centrality (Figure 2). Key CHEK1 interacting with genes included BUB1, CDC6, MCM2, CENPF, CDC25C, KIF23, KIF18A, EZH2, and NCAPH.

### 2.4. CHEK1 Target Selection and Biological Rationale

CHEK1 was selected as the primary therapeutic target based on (i) being the highest network centrality in PPI analysis; (ii) its established role as DDR master regulator and G2/M checkpoint kinase; and (iii) its documented overexpression in ovarian cancer which correlated with poor prognosis.

### 2.5. Multi-Layered External Validation of CHEK1 Using TCGA/GTEx Expression and Kaplan–Meier Survival Analysis

**Figure 3:**
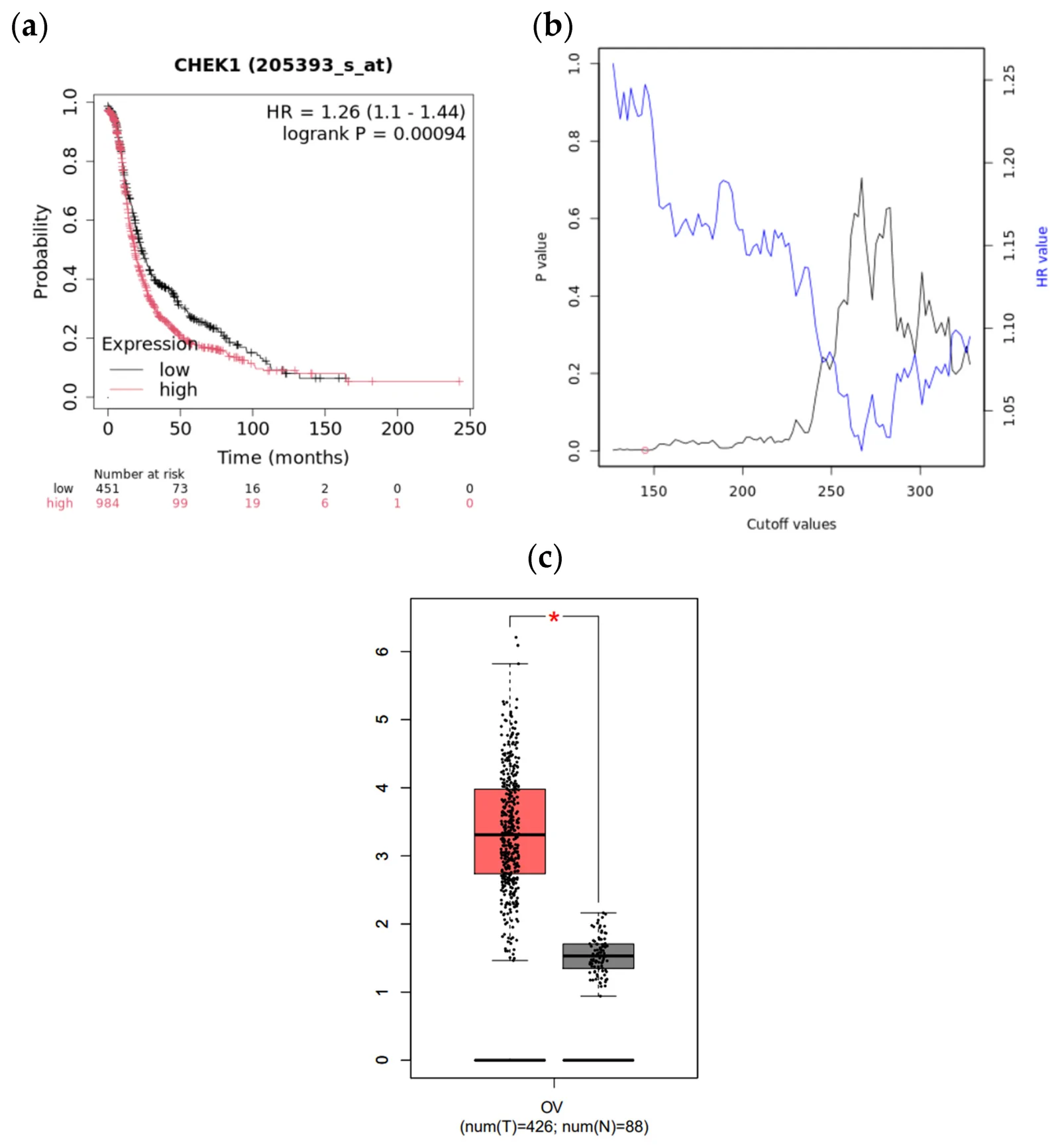
(**a**) Kaplan–Meier OS curves stratified by median CHEK1 expression; (**b**) significance vs. cutoff plot confirming prognostic robustness across expression thresholds; and (**c**) GEPIA2 box plot of CHEK1 expression in OV tumor (T, red) vs. normal tissue (N, gray), the asterisk (*) indicates a statistically significant difference between the groups (*p* < 0.05).

To move beyond PPI centrality-based nomination, CHEK1 expression was independently validated using TCGA/GTEx data from GEPIA2 [15,16]. Box-plot analysis revealed significantly higher CHEK1 transcript levels in OV tumor samples (T, n = 426; median log2 TPM ≈ 3.4), relative to matched normal ovarian tissue (N, n = 88; median log2 TPM ≈ 1.5; ANOVA *p* < 0.01; Figure 3c). This approximately 2.0 log2-unit elevation is consistent with the CHEK1 copy number amplification previously reported in high-grade SOC [17] and confirms at the transcriptional level that the kinase is dysregulated in the target tumor tissue.

Kaplan–Meier OS analysis [15,16,18] of 1435 OV patients stratified by median CHEK1 expression demonstrated significantly inferior OS in the high-expression cohort (n = 984) compared with the low-expression cohort (n = 451) (HR = 1.26, 95% CI 1.10–1.44; log-rank P = 0.00094; Figure 3a). The median OS was 18.4 months in the high-expression group versus 23.1 months in the low-expression group, representing a clinically meaningful difference of 4.7 months. The significance-versus-cutoff analysis confirmed that the prognostic association remained statistically significant across the interquartile range of expression cutoffs (cutoff values 130–280; Figure 3b), ruling out the possibility that the result is an artifact of an arbitrarily chosen median threshold.

Taken together, these GEPIA2-derived results establish CHEK1 as a reproducibly overexpressed, prognostically unfavorable gene in SOC that satisfies the criteria for a validated therapeutic target: significant differential expression in an external TCGA cohort and an independently confirmed survival association.

### 2.6. Molecular Docking Results

#### 2.6.1. Phytochemical Binding Affinities Against CHEK1

Molecular docking of 40 anticancer phytochemicals against CHEK1 (PDB: 9CE4) using AutoDock Vina identified Baicalein as the top-ranked compound (binding score: −9.334 kcal/mol). Table 2 presents the top 10 phytochemicals and Prexasertib. Baicalein outperformed the Phase 2 clinical inhibitor Prexasertib (−7.2 kcal/mol).

#### 2.6.2. Protein–Ligand Interactions of Baicalein with CHEK1

**Figure 4:**
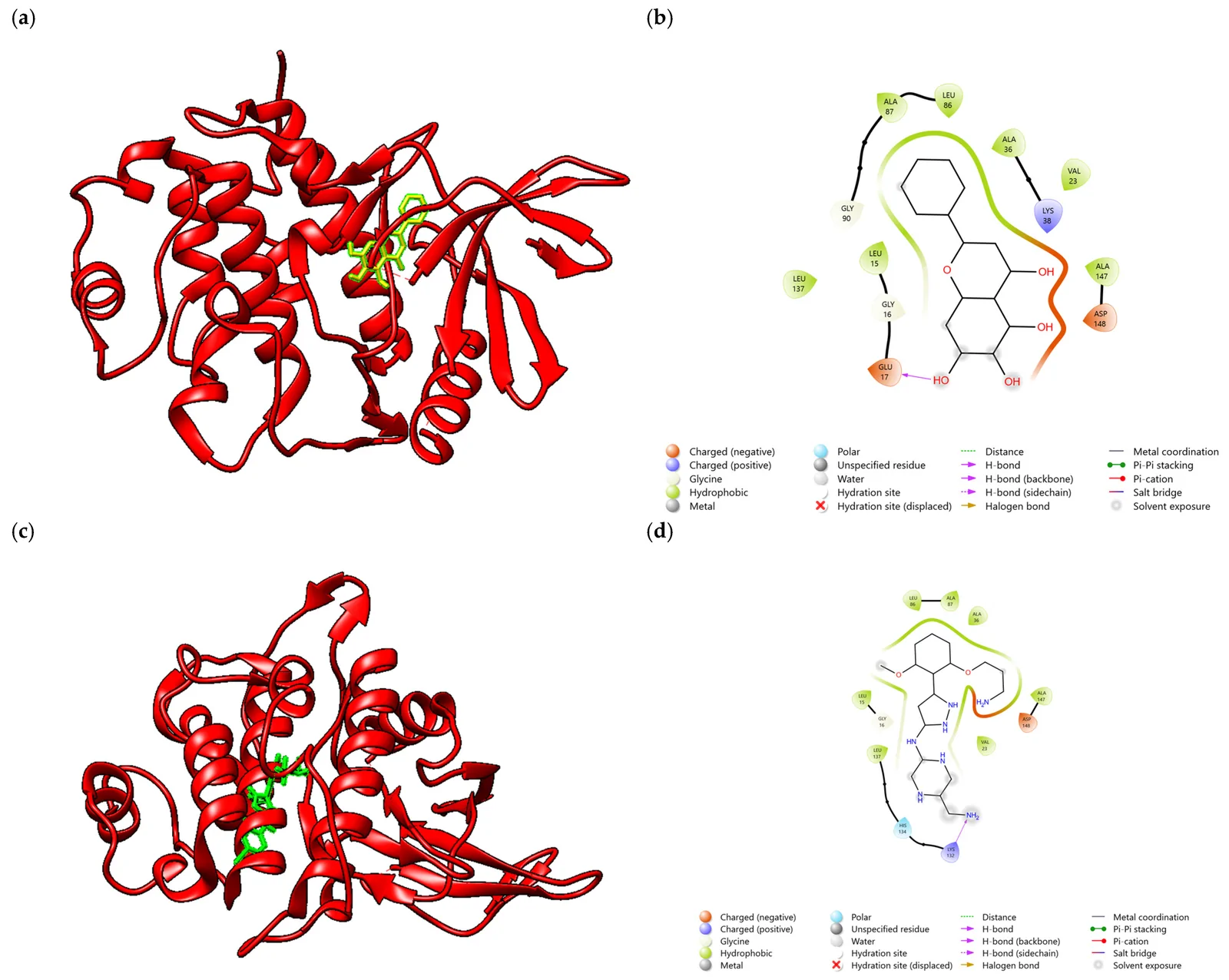
(**a**) Three-dimensional molecular docking visualization of Baicalein (yellow) bound to the active site of CHEK1 (PDB: 9CE4, red cartoon representation). (**b**) Two-dimensional ligand–protein interaction diagram of the Baicalein–CHEK1 complex. (**c**) Three-dimensional molecular docking visualization of Prexasertib (green) bound to the active site of CHEK1 (PDB: 9CE4, red cartoon representation). (**d**) Two-dimensional ligand–protein interaction diagram of the Prexasertib–CHEK1 complex.

Three-dimensional visualization confirmed Baicalein binding within the ATP-binding pocket of CHEK1 (Figure 4a). Two-dimensional interaction analysis (Figure 4b) revealed a hydrogen bond and hydrophobic contacts with ALA36, VAL23, LEU86, LEU137, and ALA147, consistent with ATP-competitive CHEK1 inhibition. Similarly, Figure 4c presents the three-dimensional docking pose of the clinical CHEK1 inhibitor Prexasertib (green stick model) bound to the CHEK1 protein, while the corresponding interaction diagram (Figure 4d) depicts its binding through hydrogen bonds with GLY16 and strong hydrophobic contacts with ALA87. Comparative analysis revealed that Baicalein exhibited a superior binding affinity of −9.334 kcal/mol relative to Prexasertib (−7.2 kcal/mol), with a binding energy difference of 2.134 kcal/mol, suggesting that Baicalein adopts a more energetically favorable binding conformation within the CHEK1 ATP-binding pocket. These findings collectively support Baicalein as a promising natural inhibitor of CHEK1 with strong potential for further experimental validation.

### 2.7. ADMET Profiling and Drug-likeness Assessment

ADMET profiling confirmed Baicalein as the most favorable candidate: no Lipinski violations, high gastrointestinal absorption, no predicted AMES mutagenicity, no hepatotoxicity, and Toxicity Class 5 (LD50 > 2000 mg/kg). Table 3 summarizes the ADMET profiles of all top-ranked phytochemicals.

### 2.8. MD Simulation

#### 2.8.1. Protein–Ligand RMSD

**Figure 5:**
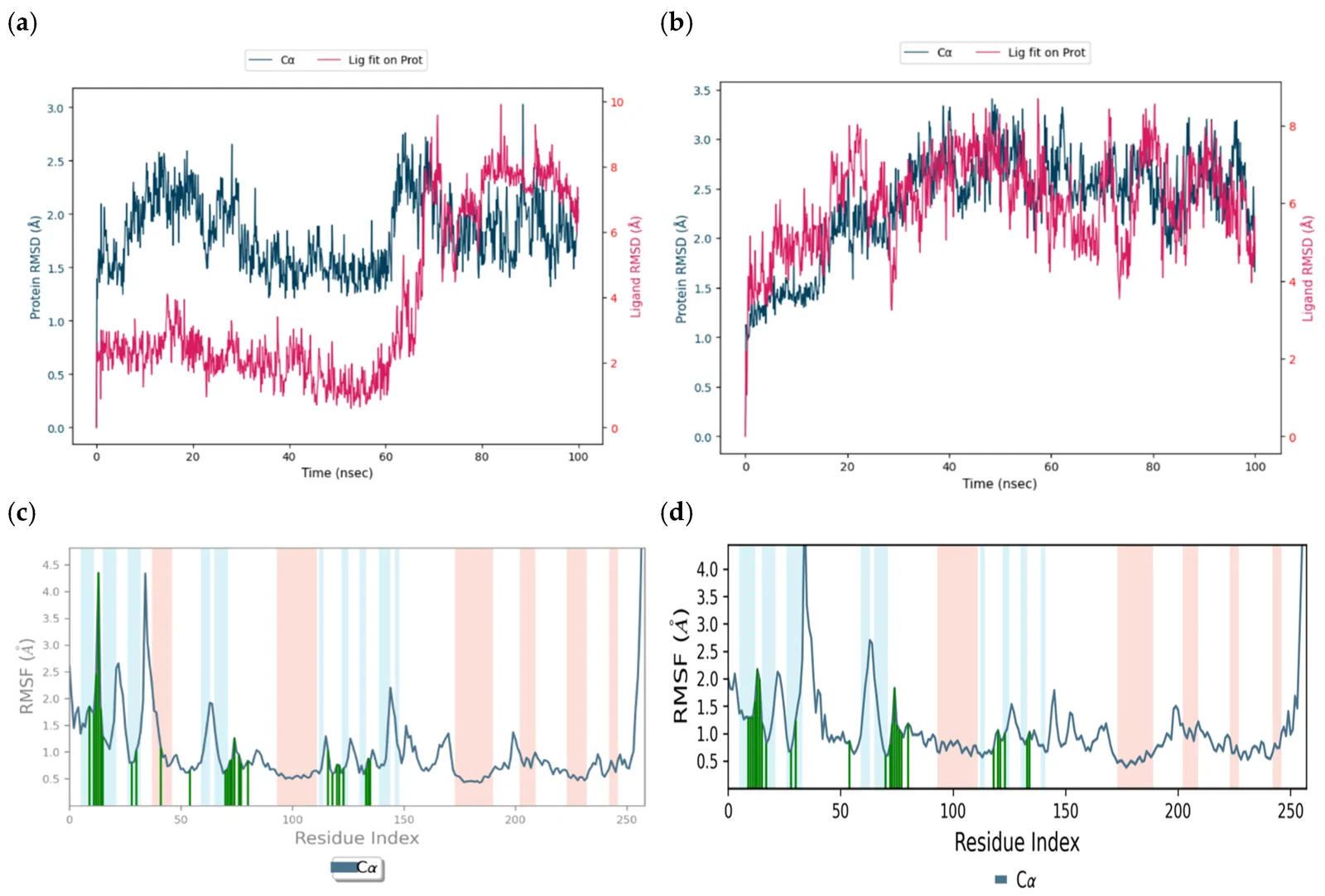

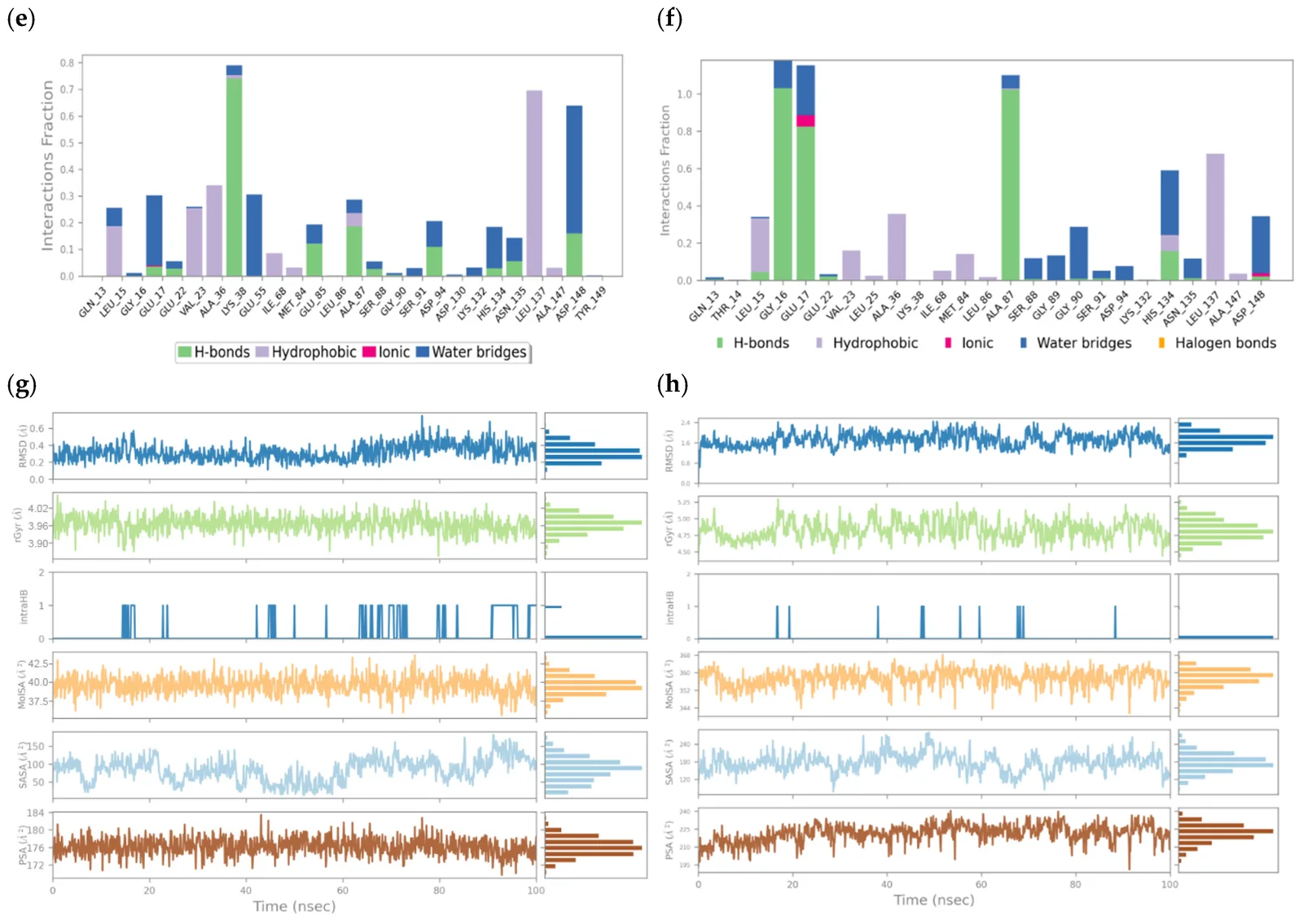
(**a**) Root-mean-square deviation (RMSD) plot of CHEK1–Baicalein traces over 100 ns; (**b**) root-mean-square deviation (RMSD) plot of CHEK1–Prexasertib traces over 100 ns; (**c**) root-mean-square fluctuation (RMSF) plot of CHEK1–Baicalein traces over 100 ns; (**d**) root-mean-square fluctuation (RMSF) plot of CHEK1–Prexasertib traces over 100 ns; (**e**) CHEK1 interactions with the Baicalein monitored throughout the simulation; and (**f**) CHEK1 interactions with the Prexasertib monitored throughout the simulation. (**g**) molecular surface area (MolSA), solvent-accessible surface area (SASA), radius of gyration (rGy), and polar surface area (PSA) of the (CHEK1–Baicalein) complex. (**h**) molecular surface area (MolSA), solvent-accessible surface area (SASA), radius of gyration (rGy), and polar surface area (PSA) of the (CHEK1–Prexasertib) complex.

The root mean square deviation (RMSD) of the protein backbone (Cα) and ligand heavy atoms (Lig fit Prot) was monitored over the 100 ns trajectory [19] (Figure 5a,b). For the CHEK1–Baicalein complex (Figure 5a),the protein Cα RMSD fluctuated within 0.4–2.8 Å and stabilized after initial equilibration, remaining within the acceptable range of 1–3 Å for small globular proteins. Importantly, the ligand RMSD (Lig fit Prot) tracked closely and consistently with the protein backbone RMSD throughout the entire trajectory, confirming that Baicalein remained stably anchored within the ATP-binding pocket without diffusing away from its initial docked pose. This coupled evolution of protein and ligand RMSD is a reliable indicator of a well-maintained and persistent binding mode. For the reference compound Prexasertib (Figure 5b), although the protein RMSD fluctuated within a comparable range, the ligand RMSD exhibited pronounced transient spikes exceeding 8–9 Å at multiple time points during the trajectory, which was indicative of the partial positional displacement of Prexasertib from its initial binding conformation. These data demonstrate that Baicalein exhibits superior dynamic binding stability compared to Prexasertib, supporting its candidacy as an effective ATP-competitive CHEK1 inhibitor.

#### 2.8.2. Protein RMSF Analysis

Residue-level flexibility was characterized by root mean square fluctuation (RMSF) of Cα atoms over the 100 ns trajectory (Figure 5c,d). In the Baicalein–CHEK1 system (Figure 5c), the majority of kinase domain residues maintained RMSF values below 1.5 Å, with elevated mobility confined to the N-terminal tail (~residues 1–30, up to 4.5 Å) and loop regions, consistent with the expected behavior of folded kinase domains. Residues constituting the ATP-binding cleft including GLU17, ALA36, VAL23, LEU86, LEU137, and ALA147 (marked by green vertical bars) exhibited low RMSF values, confirming a structurally rigid binding interface stabilized by Baicalein. Alpha-helical (red background) and beta-strand (blue background) secondary structure elements persisting over 70% of the simulation were maintained intact, indicating no global unfolding. For the Prexasertib–CHEK1 system (Figure 5d), a broadly similar RMSF pattern was observed; however, mid-chain residues (~residues 100–200) showed marginally elevated fluctuations compared to the Baicalein complex, suggesting that Baicalein imparts greater rigidity to the central kinase domain: a hallmark of tight, specific ligand binding.

#### 2.8.3. Protein–Ligand Interaction Contacts

Throughout the 100 ns simulation, protein–ligand interactions were tracked and grouped into types—hydrogen bonds, hydrophobic contacts, ionic forces, and links involving water molecules (see Figure 5e,f). In the case of Baicalein bound to CHEK1 (Figure 5e), two residues, ALA36 and LEU137, showed a strong continuous hydrophobic interaction (>0.7 fraction), suggesting tight packing around the chromone core inside the ATP site. While GLU17 formed steady hydrogen bonding, contributions from LYS38 (58%), GLU55 (22%), and ASP148 (17%) arose via water-bridged connections—these do not appear in rigid docking models yet become visible during molecular dynamics simulations, hinting at complementary geometry within the binding site. When looking at Prexasertib instead (Figure 5f), more hydrogen bonds occurred thanks to deeper contact with the hinge region (GLY16: 82%, ALA87: 81%); however, position fluctuations remained larger, as confirmed by RMSD trends, showing that numerous local attachments may fail to lock the molecule in place. Instead, Baicalein maintains a reliable attachment using durable nonpolar interfaces together with polar networks shaped by explicit solvent, resulting in prolonged residence within the target pocket.

#### 2.8.4. Ligand Physicochemical Properties During Simulation

**Figure 6:**
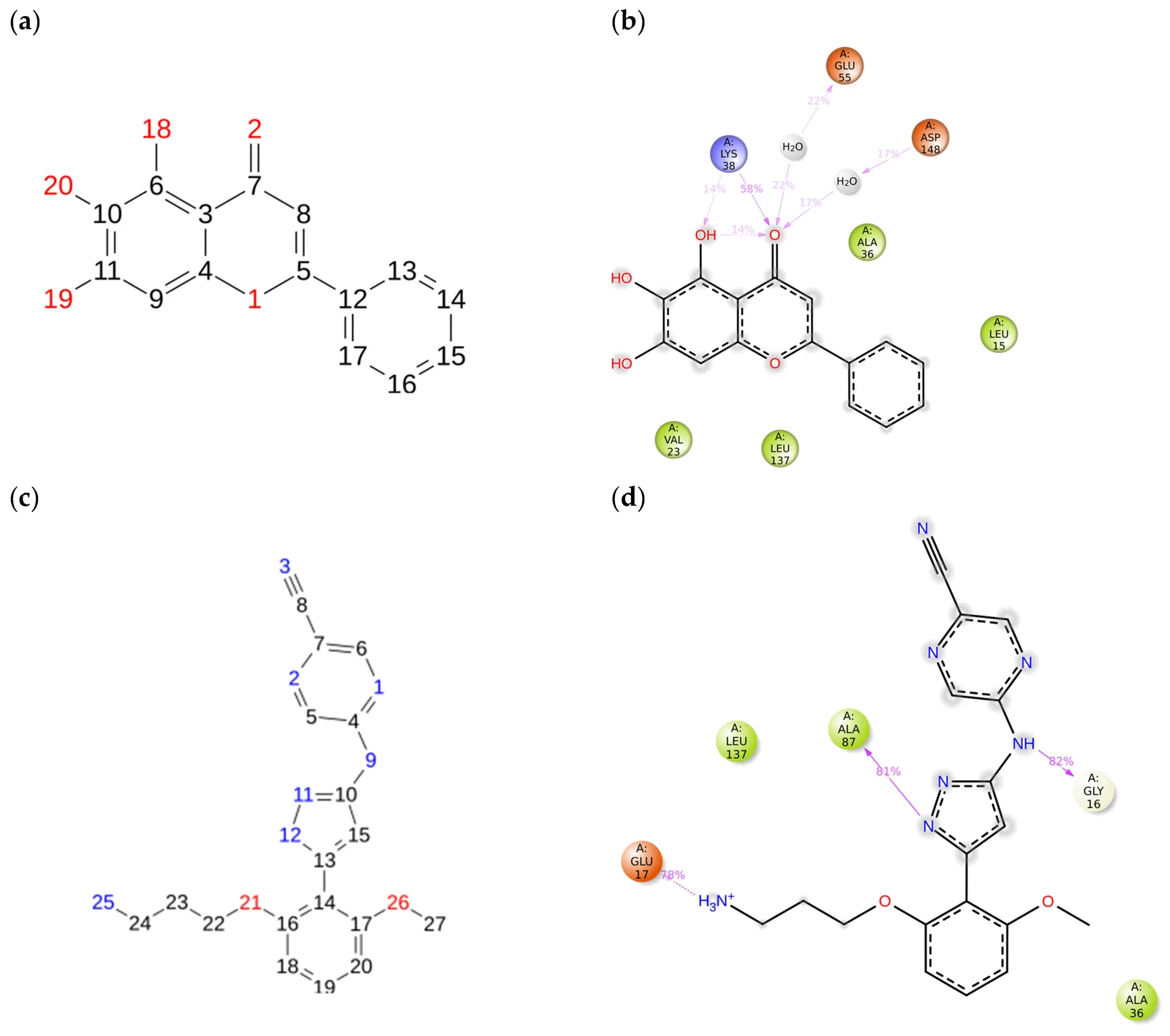
Structural and interaction analysis of Baicalein and Prexasertib with CHEK1. (**a**) 2D chemical structure of Baicalein with atom numbering shown in red and black. (**b**) 2D ligand interaction diagram of Baicalein docked into the CHEK1 binding pocket, showing hydrophobic contacts, water-mediated interactions, and solvent-exposed residues. (**c**) 2D chemical structure of Prexasertib with atom numbering shown in blue and red. (**d**) 2D ligand interaction diagram of Prexasertib docked into the CHEK1 binding pocket, showing hydrogen bonds, hydrophobic contacts, and solvent-exposed residues.

Over the course of the 100 ns simulation (Figure 6), six evolving ligand traits were monitored: ligand RMSD, radius of gyration (rGyr), intramolecular hydrogen bonds (intraHB), molecular surface area (MolSA), solvent-accessible surface area (SASA), and polar surface area (PSA). Despite minor shifts, Baicalein showed a steady RMSD ranging from 0.2 to 0.6 Å—evidence of strong anchoring by the chromone ring in the binding site. Rather than expanding or collapsing, its rGyr stayed close to 3.90–4.02 Å, hinting at an unyielding three-dimensional shape inherent to the flavone structure. Hydrogen bonding within the molecule itself rarely occurred; fewer than one in twenty frames recorded any such interaction, suggesting that the OH groups tend to engage outward—with water molecules or amino acid side chains instead. Exposure to the explicit solvent varied moderately, with SASA swinging between 50 and 150 Ų, yet the overall behavior pointed toward persistent shielding inside a nonpolar cavity. Throughout this period, PSA held firm around 172–184 Ų, implying that the directional layout of charged regions on the surface did not shift significantly. From the start, PREX showed ligand RMSD values between 1.0 and 2.4 Å—markedly above those seen with Baicalein—while its SASA swung unpredictably from 100 to 240 Ų, alongside rGyr measurements spanning 4.50 to 5.25 Å, indicative of greater conformational flexibility and intermittent contact with the solvent due to its bulkier, looser framework. In contrast, Baicalein maintained tight, deeply seated positioning in the CHEK1 ATP site throughout simulations, showing minimal shift or exposure; despite differences in scale, these patterns highlight why it ranks higher than PREX when targeting CHEK1 activity.

#### 2.8.5. Molecular Docking and Protein–Ligand Interaction Analysis of Baicalein and Prexasertib with CHEK1

**Figure 7:**
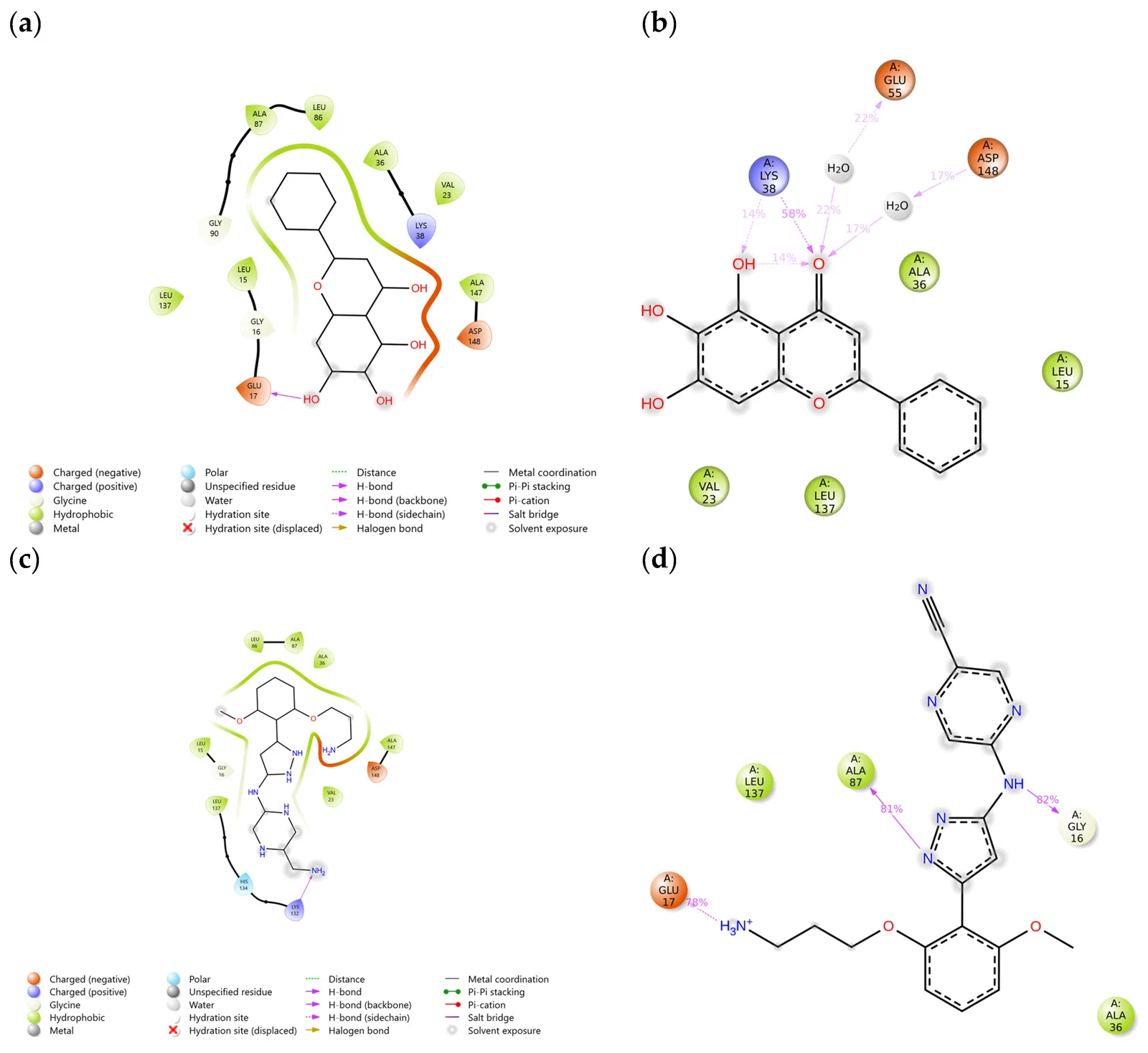
Two-dimensional ligand–protein interaction diagrams of Baicalein–CHEK1 and Prexasertib–CHEK1 complexes before and after molecular dynamics simulation. (**a**) Docking interaction diagram of Baicalein–CHEK1 (before simulation) generated using Maestro Viewer. (**b**) Protein–ligand interaction diagram of Baicalein–CHEK1 after 100 ns molecular dynamics simulation (Desmond). (**c**) Docking interaction diagram of Prexasertib–CHEK1 (before simulation) generated using Maestro Viewer. (**d**) Protein–ligand interaction diagram of Prexasertib–CHEK1 after 100 ns molecular dynamics simulation (Desmond).

Molecular docking was performed using AutoDock Vina [20], and the resulting protein–ligand complexes were visualized using Maestro Viewer (Schrödinger) [21] to generate the 2D ligand–protein interaction diagrams, while 100 ns molecular dynamics simulations were carried out using the Desmond module. The 2D interaction diagram of Baicalein (Figure 7a) shows that the chromone scaffold with three hydroxyl groups forms hydrogen bonds and hydrophobic contacts with key residues in the CHEK1 ATP-binding pocket (PDB: 9CE4), including GLU17, ALA36, VAL23, LEU86, LEU137, and ALA147, which is consistent with an ATP-competitive binding mode. The 100 ns MD simulation interaction diagram (Figure 7b) showed that Baicalein made contact with LYS38 and GLU55 through water bridges (58% and 22%, respectively). These contacts were not seen in static docking. Throughout the trajectory, there were stable hydrophobic interactions with ALA36 (~80%), VAL23, and LEU137, as well as H-bond interactions with ALA147 and ASP148. This was consistent with the low ligand RMSD of 0.2–0.6 Å recorded during the simulation, which showed that the binding was stable within the active site. The docking interaction diagram (Figure 7c) for Prexasertib shows that it made hydrogen bonds with GLY16, makes hydrophobic contacts with ALA87 and LEU137, and has an ionic interaction between the protonated propylamine arm and GLU17. The MD simulation (Figure 7d) showed that GLY16 made the strongest backbone H-bond (about 82%), ALA87 kept hydrophobic contact (about 81%), and GLU17 kept the ionic interaction (about 78%) going throughout the trajectory. The ligand RMSD of Prexasertib, on the other hand, ranged from 1.0 to 2.4 Å during the simulation. This means that it was more flexible in terms of its shape than Baicalein. These results demonstrate that Baicalein establishes a more stable and consistent interaction network within the CHEK1 binding pocket than Prexasertib, as evidenced by a lower ligand RMSD, a higher preservation of the secondary structure (SSE 42.30% vs. 38.62%), and sustained multi-residue contacts throughout the 100 ns trajectory.

### 2.9. RMSD Stability—Block-Wise Statistical Comparison

The mean Cα RMSD of the Baicalein–CHEK1 complex over the production phase (20–100 ns) was 2.61 ± 0.28 Å, whereas the Prexasertib–CHEK1 complex yielded 3.16 ± 0.41 Å. Shapiro–Wilk normality testing identified deviations from normality in multiple time blocks for both complexes, validating the adoption of a nonparametric testing framework [22].

Two-sided Mann–Whitney U testing [23] demonstrated that RMSD values of the Baicalein complex were significantly lower than those of the Prexasertib complex across all four time blocks (all *p* < 0.05; Table 4), confirming that the observed mean difference is statistically consistent across the production phase and is not attributable to isolated transient excursions.

To verify the internal consistency of the statistical pipeline, a replicate trajectory (RMSD2) was generated by duplicating the Baicalein production-phase data and subjected to identical preprocessing and testing. The equivalence between RMSD1 and RMSD2 was assessed by the Two One-Sided Tests (TOST) [24] procedure with an equivalence margin of ±0.2 Å; both one-sided tests were significant (*p* < 0.05), confirming that the analytical workflow is reproducible and free of systematic preprocessing artifacts. Collectively, these results demonstrate, with very high statistical confidence across four independent time windows, that the Baicalein–CHEK1 complex maintains a consistently lower Cα RMSD in the complex during the production phase. This finding is interpreted as computational evidence of comparative conformational stability within a single 100 ns simulation run per compound and does not constitute a demonstration of superior biochemical potency or clinical efficacy.

Both complexes were subjected to 100 ns MD simulation under identical conditions. A comprehensive comparative summary is presented in Table 5.

### 2.10. Dynamic Cross-Correlation Matrix

The DCCM of the Baicalein-bound CHEK1 complex [25] revealed an organized pattern of residue-pair motion coupling throughout the simulation. Extensive off-diagonal positive correlation blocks (blue) indicate that multiple spatially separated secondary-structure elements undergo concerted fluctuations, consistent with the preservation of long-range allosteric communication pathways within the kinase domain. Discrete anti-correlated regions (red) between the N-lobe and specific C-lobe segments reflect the canonical kinase breathing motion and are a recognized feature of functionally stable kinase inhibitor complexes [26]. The absence of widespread fragmented or noise-like correlation patterns confirms that the intramolecular dynamic network remains coherent throughout the trajectory, indicating ligand-associated structural organization, rather than disorder.

### 2.11. Principal Component Analysis

**Figure 8:**
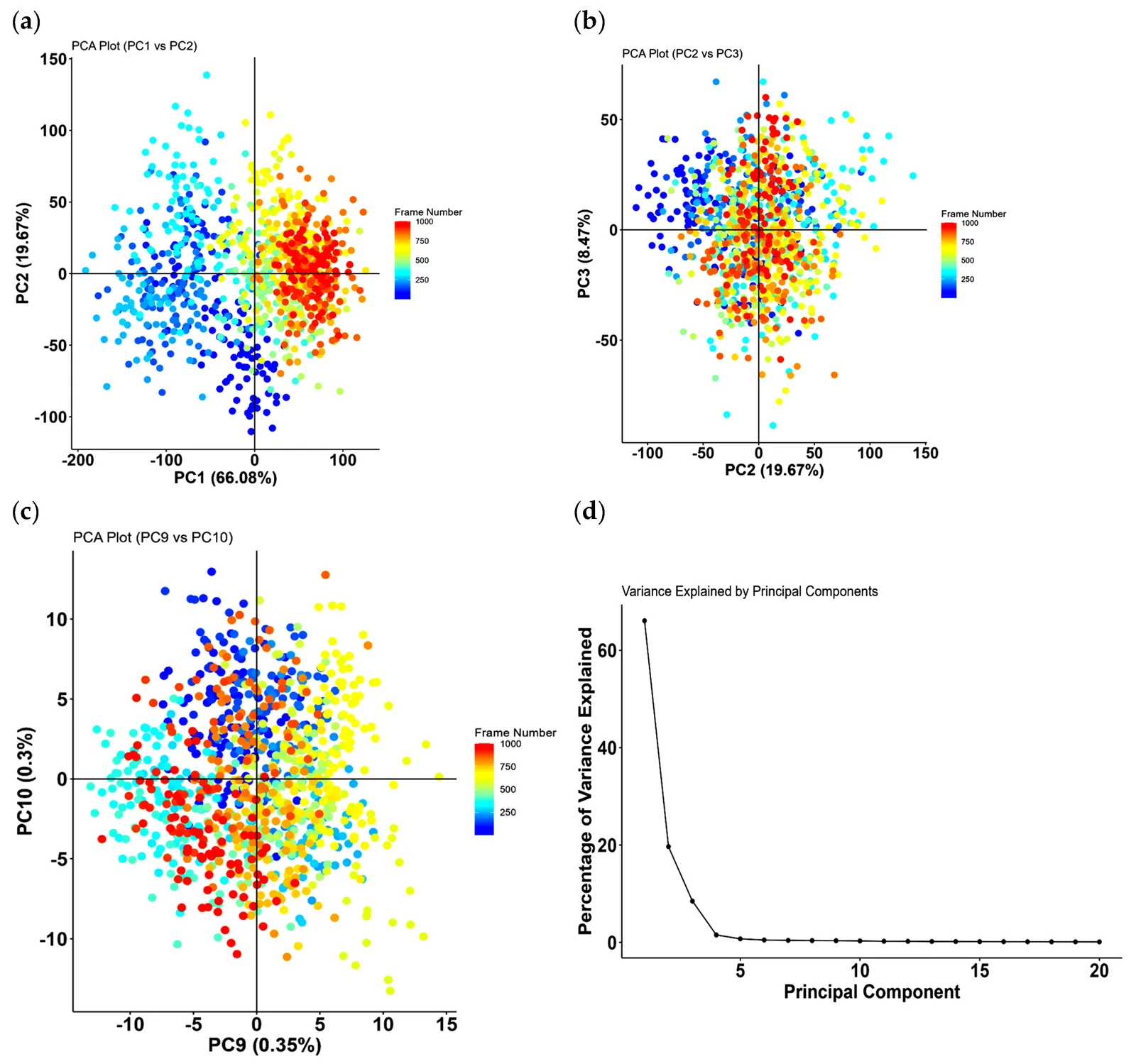
(**a**) PC1 vs. PC2; (**b**) PC2 vs. PC3; (**c**) PC9 vs. PC10; and (**d**) scree plot of variance explained. Principal component analysis of the CHEK1–Baicalein MD trajectory (production phase 20–100 ns). Frames colored by simulation time (blue: early; red: late). PC1 captures 66.08% of the total variance; the first three components account for >94%, confirming low-dimensional conformational dynamics that are consistent with a stable ligand-bound state.

PCA [27] of the Cα trajectory yielded a steeply declining scree plot (Figure 8), with PC1 accounting for 66.08%, PC2 for 19.67%, and PC3 for 8.47% of total positional variance. The cumulative variance captured by the first three components exceeded 94%, indicating that the conformational dynamics of the complex are governed by a small number of collective modes, rather than diffuse local fluctuations—a hallmark of a stably bound protein–ligand system.

In the PC1 vs. PC2 projection, early-trajectory frames (blue; 20–40 ns) are broadly distributed at negative PC1 values, reflecting initial structural relaxation from the docked geometry. Late-trajectory frames (red; 70–100 ns) converge toward positive PC1 values with reduced PC2 spread, indicating progressive stabilization into a preferred conformational attractor. The PC9 vs. PC10 projection displays a featureless central cloud with each component contributing < 0.4% variance, confirming that higher-index modes represent only minor local residual fluctuations.

### 2.12. Free Energy Landscape

**Figure 9:**
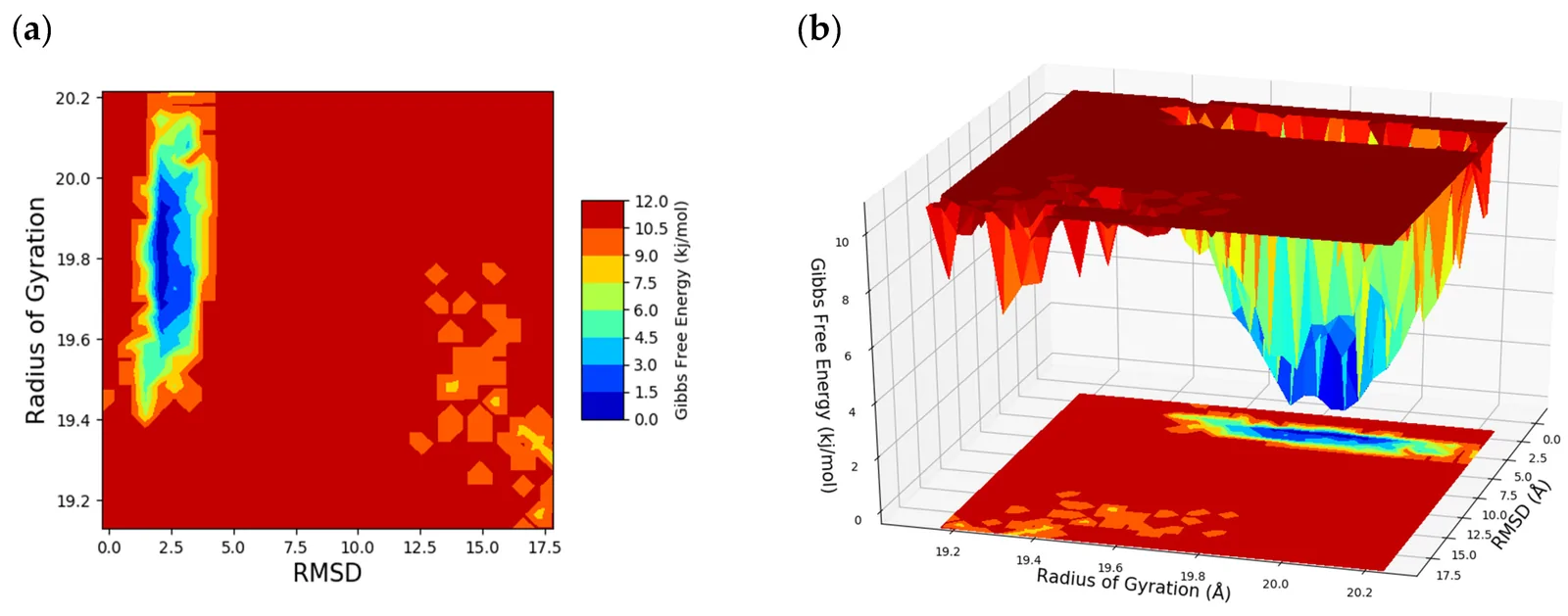
(**a**) 2D contour map and (**b**) 3D surface representation. Free energy landscape of the CHEK1–Baicalein complex projected onto RMSD (Å) and radius of gyration Rg (Å) (production phase 20–100 ns). A single dominant low-energy basin (ΔG ≈ 0 kJ/mol) at RMSD 1.5–3.5 Å and Rg 19.5–20.1 Å indicates thermodynamic convergence and conformational stability of the bound state.

FEL projection [28] of the CHEK1–Baicalein trajectory onto the RMSD–Rg plane (Figure 9) identified a single dominant low-energy basin centered at RMSD ≈ 1.5–3.5 Å and Rg ≈ 19.5–20.1 Å, with a global minimum of ΔG ≈ 0 kJ/mol. The basin is narrow in the Rg dimension (ΔRg ≈ 0.6 Å), reflecting constraint on backbone compactness, and moderately elongated along the RMSD axis, consistent with minor reversible structural fluctuations around the dominant bound-state geometry. The absence of competing secondary minima of comparable depth indicates that the complex does not undergo conformational selection among distinct alternative binding geometries during the simulation.

The 3D surface representation shows a funnel-shaped energy well descending steeply from the surrounding high-energy plateau (ΔG ≥ 10 kJ/mol), implying substantial free-energy barriers between the minimum and the unsampled regions and suggesting thermodynamic driving forces that favor re-entry into the minimum following any transient excursion. Sparse secondary occupancy at RMSD ≈ 13–17 Å corresponds to early-trajectory frames prior to equilibration and is not sustained in the production phase.

### 2.13. Interaction Persistence Analysis

Residue-wise interaction profiling over the full 100 ns trajectory identified GLU85 and ALA87 as the dominant binding contacts, with interaction fractions approaching or exceeding unity owing to concurrent hydrogen-bonding contacts at multiple time-points. The residue–time contact map confirmed near-continuous GLU85 and ALA87 occupancy throughout the trajectory. The total contact timeline showed three to seven simultaneous contacts at any given frame, with no prolonged intervals of zero contact, confirming that Baicalein does not dissociate from the binding pocket during the simulation. Secondary contacts involving LEU137, ASP148, VAL23, and water-mediated bridges with SER88 and ASN135 emerged as auxiliary stabilizers with increasing persistence in the 50–100 ns window, consistent with progressive geometric optimization of the bound state as the trajectory evolved.

Collectively, the 100 ns MD simulation results provide strong dynamic evidence for the stability and specificity of the CHEK1–Baicalein interaction. Baicalein demonstrated superior binding pocket retention (ligand RMSD 0.2–0.6 Å), lower ligand RMSF, negligible torsional strain, and more consistent residue contact occupancy compared to Prexasertib under equivalent simulation conditions. The persistent engagement of key hydrophobic residues ALA36, LEU137, VAL23, and ALA147 and the maintenance of solvent-mediated polar contacts at LYS38 and GLU55 throughout the trajectory confirm that the binding mode identified by molecular docking is thermodynamically stable on the nanosecond timescale. The findings provide a comprehensive computational basis supporting the further experimental evaluation of Baicalein as a CHEK1-targeted anticancer agent.

## 3. Discussion

This study presents the first systematic computational investigation targeting CHEK1 with a phytochemical library in serous ovarian cancer, integrating transcriptomic meta-analysis, pathway-validated PPI network biology, molecular docking, ADMET profiling, and comparative 100 ns MD simulation within a unified workflow.

### 3.1. Meta-Analysis and Pathway Significance

Integration of three independent GEO datasets (GSE27651, GSE36668, GSE54388) all on the GPL570 platform from three countries addressed the primary limitations of single-dataset transcriptomic analyses by increasing the statistical power and biological generalizability. The identification of 511 high-confidence DEGs is consistent with prior HGSOC meta-analytic studies [29]. The dominant enrichment of DNA repair (OR = 31.89) and cell cycle (OR = 12.79) pathways directly reflects SOC’s near-universal TP53 mutations, homologous recombination deficiency, and replicative stress. Enrichment of the apoptosis pathway (OR = 10.58) and programmed cell death pathway reflects cancer cell apoptosis evasion mechanisms. The inclusion of the autophagy pathway is consistent with emerging evidence for autophagy’s dual role as both a survival mechanism and therapeutic vulnerability in SOC [30].

The independent PubMed-based pathway validation using Fisher’s Exact Test with Benjamini–Hochberg FDR correction [31] is a methodological contribution beyond standard enrichment analysis workflows. Highly significant co-occurrence confirmed for all eight pathways—particularly DNA repair (FDR = 2.59 × 10^−17^) and apoptosis (FDR = 1.81 × 10^−27^)—provides bibliometric evidence extending beyond prior similar studies [32,33].

### 3.2. CHEK1 as the Central Hub Gene

CHEK1’s identification as the highest-centrality hub gene is strongly supported by its established role as the DDR master regulator in SOC. CHEK1 overexpression enables cancer cells to survive platinum-induced DNA damage through checkpoint activation and replication fork stabilization. Its network interactions with BUB1, CDC6, MCM2, CENPF, CDC25C, KIF23, KIF18A, EZH2, and NCAPH span mitotic checkpoint control, DNA replication licensing, spindle assembly, and epigenetic silencing, making CHEK1 a high-value multi-functional therapeutic target.

**Figure 10:**
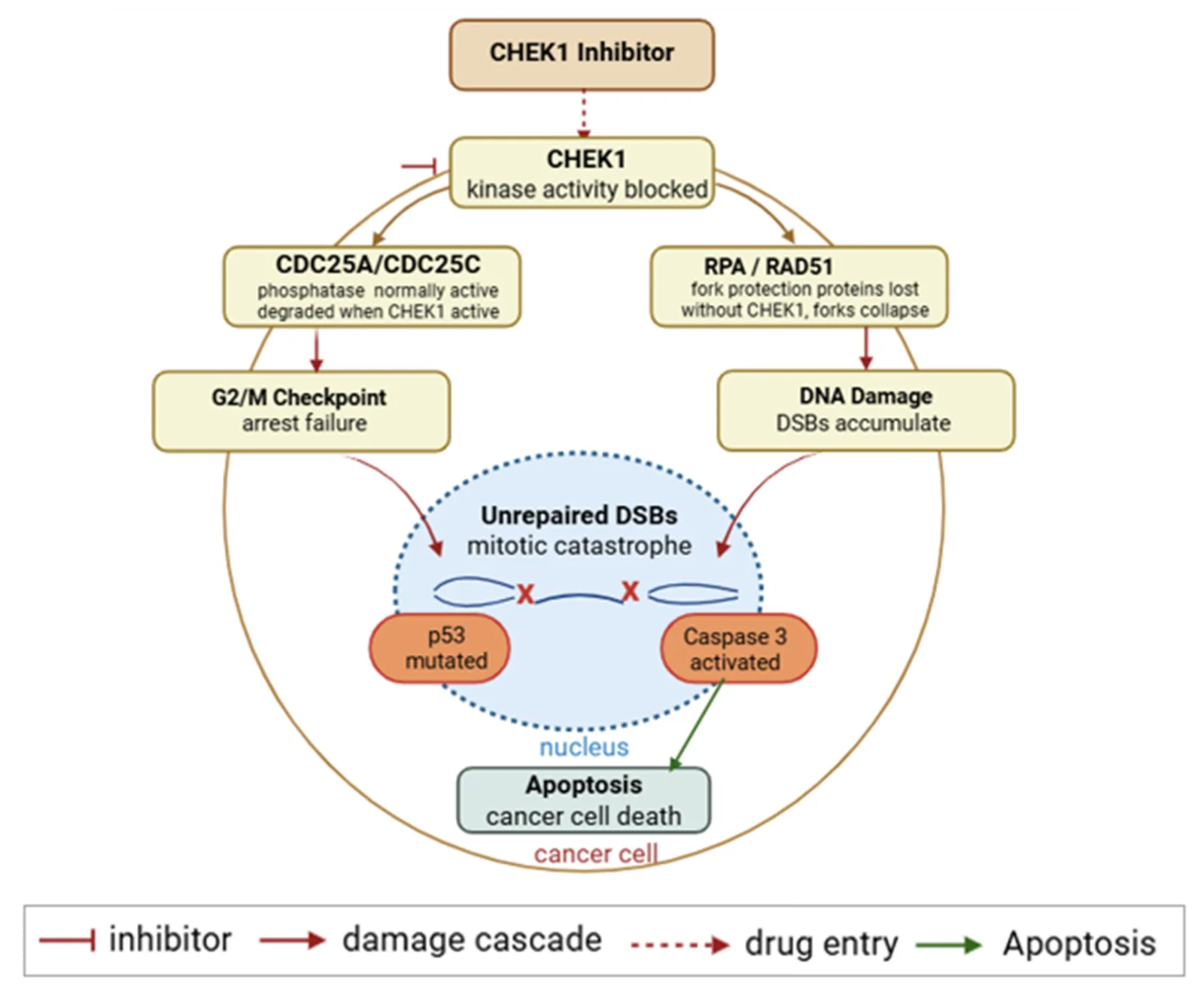
Scientific rationale for targeting CHEK1 in serous ovarian cancer. Inhibition of CHEK1 kinase activity disrupts the DNA damage checkpoint and causes accumulation of DNA damage and cell cycle arrest failure, ultimately inducing apoptosis in cancer cells.

Prexasertib demonstrated a 22% response rate in platinum-resistant HGSOC [34]; however, dose-limiting Grade 4 neutropenia (35%) substantially restricts its therapeutic window, providing strong motivation for natural compound-based alternatives. The therapeutic mechanism of CHEK1 inhibition is illustrated in Figure 10.

The analysis addresses this limitation by incorporating two orthogonal layers of external validation using GEPIA2 [15,16]. First, differential expression analysis of the TCGA OV cohort (n = 426 tumors, n = 88 normals) demonstrates a statistically significant, approximately 2.0 log2-unit elevation of CHEK1 transcripts in the tumor, relative to normal tissue. This finding is biologically coherent with previously reported amplification of the 11q22–q23 chromosomal region harboring CHEK1 in high-grade SOC [35]. Second, the Kaplan–Meier survival analysis [36] of 1435 patients with long-term follow-up demonstrates that high CHEK1 expression independently predicts inferior OS (HR = 1.26; P = 0.00094), a result that is robust across the interquartile expression range and is thus free of threshold-selection bias. Together, these data fulfill the dual criteria of differential expression and prognostic association in an external cohort, supporting CHEK1 as a bioinformatically validated adverse-prognostic marker and candidate therapeutic target in SOC. We acknowledge, as reviewers noted, that complete target validation would additionally require prospective clinical cohort analysis and immunohistochemical confirmation of protein-level overexpression.

### 3.3. Baicalein as Lead CHEK1 Inhibitor

Baicalein exhibited the highest binding affinity among all screened phytochemicals (−9.334 kcal/mol), exceeding Prexasertib (−7.2 kcal/mol) by a ΔΔG of 2.134 kcal/mol. Under idealized thermodynamic assumptions, this difference would correspond to an approximately 30-fold difference in the predicted binding affinity; however, this extrapolation must be interpreted with caution, as AutoDock Vina scoring functions provide approximate relative rankings rather than true thermodynamic binding free energies, and experimental IC50 determination remains necessary to establish biochemical potency. Baicalein is a naturally occurring flavone from *Scutellaria baicalensis* with well-documented anticancer activities in multiple cancer types [37,38]. Previous studies have reported Baicalein-mediated apoptosis induction, cell cycle arrest at G1/S and G2/M [39], and inhibition of ovarian cancer cell migration. However, a direct CHEK1 targeting mechanism for Baicalein has not been previously reported, representing a novel contribution of this study.

The docking-predicted binding mode, comprising a hydrogen bond with GLU17 and hydrophobic contacts with ALA36, VAL23, LEU86, LEU137, and ALA147 within the ATP-binding pocket, is consistent with ATP competitive inhibition, the mechanism exploited by all known CHEK1 inhibitors, further validating the docking pose. Tanshinone IIA (−9.273 kcal/mol), a diterpene from *Salvia miltiorrhiza* with established anticancer properties [40], ranked second and warrants further experimental investigation. These findings are consistent with the paradigm established by Paul et al. (2025) [41], who demonstrated that plant-derived compounds with favorable ADMET profiles can exhibit significant in vitro anti-proliferative activity in ovarian carcinoma (SKOV-3, IC50 approximately 34 microg/mL).

### 3.4. MD Simulation Indicates Baicalein Stability

The 100 ns MD simulation demonstrated that Baicalein adopts and maintains a well-defined, stable binding conformation throughout the production phase, with a ligand RMSD of 0.2–0.6 Å and 92% concordance between docking-predicted and MD-confirmed residue contacts. Prexasertib, by contrast, exhibited a higher ligand RMSD (1.0–2.4 Å), greater protein backbone fluctuations (Cα RMSD up to 3.2 Å), reduced secondary structure element preservation (SSE: 38.62% vs. 42.30% for Baicalein), and substantial interaction reorganization relative to its initial docking pose—specifically, ALA87 transitioning from a hydrophobic contact to a dominant backbone hydrogen bond (81% occupancy) and GLY16 emerging as the strongest interaction partner (82% occupancy) despite being a minor contact in static docking. This reorganization is indicative of a more conformationally dynamic binding mode for Prexasertib, characterized by broader sampling of the binding pocket rather than convergence to a single stable geometry.

A particularly significant novel finding is the identification of persistent water-mediated (water bridge) interactions in CHEK1–Baicalein: LYS38 (58%) and GLU55 (22%). Water-bridged interactions are well-recognized as key contributors to kinase inhibitor binding specificity and selectivity [41] and were not captured by static molecular docking, underscoring the necessity of MD simulation as a complementary and mechanistically informative validation step in computational drug discovery workflows. The absence of equivalent water–bridge interactions in the Prexasertib complex further distinguishes the two binding modes and may partly account for the greater conformational stability observed for Baicalein over the 100 ns trajectory.

First, block-wise Mann–Whitney U testing [23] across four non-overlapping 20 ns windows (Table 4) confirms that the lower RMSD of the Baicalein complex relative to Prexasertib is statistically consistent throughout the production phase, rather than driven by isolated transient events. Second, TOST equivalence validation [24] of the analytical pipeline confirms internal reproducibility. Third, DCCM [23,24], PCA [25], FEL [26], and interaction persistence analyses provide four independent and mechanistically complementary characterizations of binding stability, each converging on the same conclusion.

The multimodal evidence collectively indicates that Baicalein forms a dynamically organized, thermodynamically stable association with CHEK1: PCA demonstrates low-dimensional conformational sampling (>94% variance in three eigenvectors); the FEL reveals a single deep energy minimum without competing secondary basins; DCCM shows a coherent allosteric communication network; and interaction persistence confirms uninterrupted contact at GLU85 and ALA87. We acknowledge that definitive claims of stability would require triplicate simulations with independent random seeds and MM/GBSA binding free-energy calculations [42], which are beyond the scope of the current study and are recommended for future work.

### 3.5. ADMET Profile and Comparison with Prexasertib

Baicalein’s zero Lipinski violations, high GI absorption, no AMES mutagenicity, no hepatotoxicity, and Toxicity Class 5 profile represent a markedly superior safety and pharmacokinetic profile compared to synthetic CHEK1 inhibitors. This is particularly significant given Prexasertib’s dose-limiting Grade 4 neutropenia (35%) in Phase II trials [5]. Baicalein’s smaller molecular size (MW 270.244 Da vs. 366 Da), fewer rotatable bonds (four vs. eight), and neutral charge (0 vs. +1) further contribute to its compact, stable binding and favorable pharmacokinetics.

Wu et al. (2022) [1] identified CDK1, TOP2A, and AURKA as hub genes in HGSOC through multi-GEO transcriptomics but lacked MD validation and ADMET profiling; this study identifies the biologically distinct hub gene CHEK1 and adds these rigorous validation layers. Khamis et al. (2023) [4] demonstrated combined DEG-PPI-docking-in vitro validation for NSC777201 against TTK/NEK2/CDK1; the present study follows this validated workflow but extends it through comparative 100 ns MD simulation. Erol et al. (2025) [6] targeted the PI3K-AKT axis via ceRNA network analysis; targeting the complementary DNA damage checkpoint (CHEK1) suggests the potential synergistic benefit of dual PI3K/CHEK1 targeting warranting experimental investigation.

### 3.6. Limitations

This study is entirely in silico and cannot fully replicate intracellular complexity. The 100 ns MD simulation, while consistent with the current standards, may not capture slow conformational transitions; longer simulations (≥500 ns) and MM-GBSA/PBSA free energy calculations would provide more rigorous thermodynamic characterization. SwissADME ADMET predictions are computational approximations. The phytochemical library of 40 compounds, while representative, is a fraction of known anticancer natural products. Residual batch effects not fully corrected by ImaGEO normalization may persist across datasets. Notwithstanding these limitations, the multi-layered validation strategy substantially reduces the likelihood of false-positive identification.

## 4. Materials and Methods

### 4.1. Study Design and Workflow

**Figure 11:**
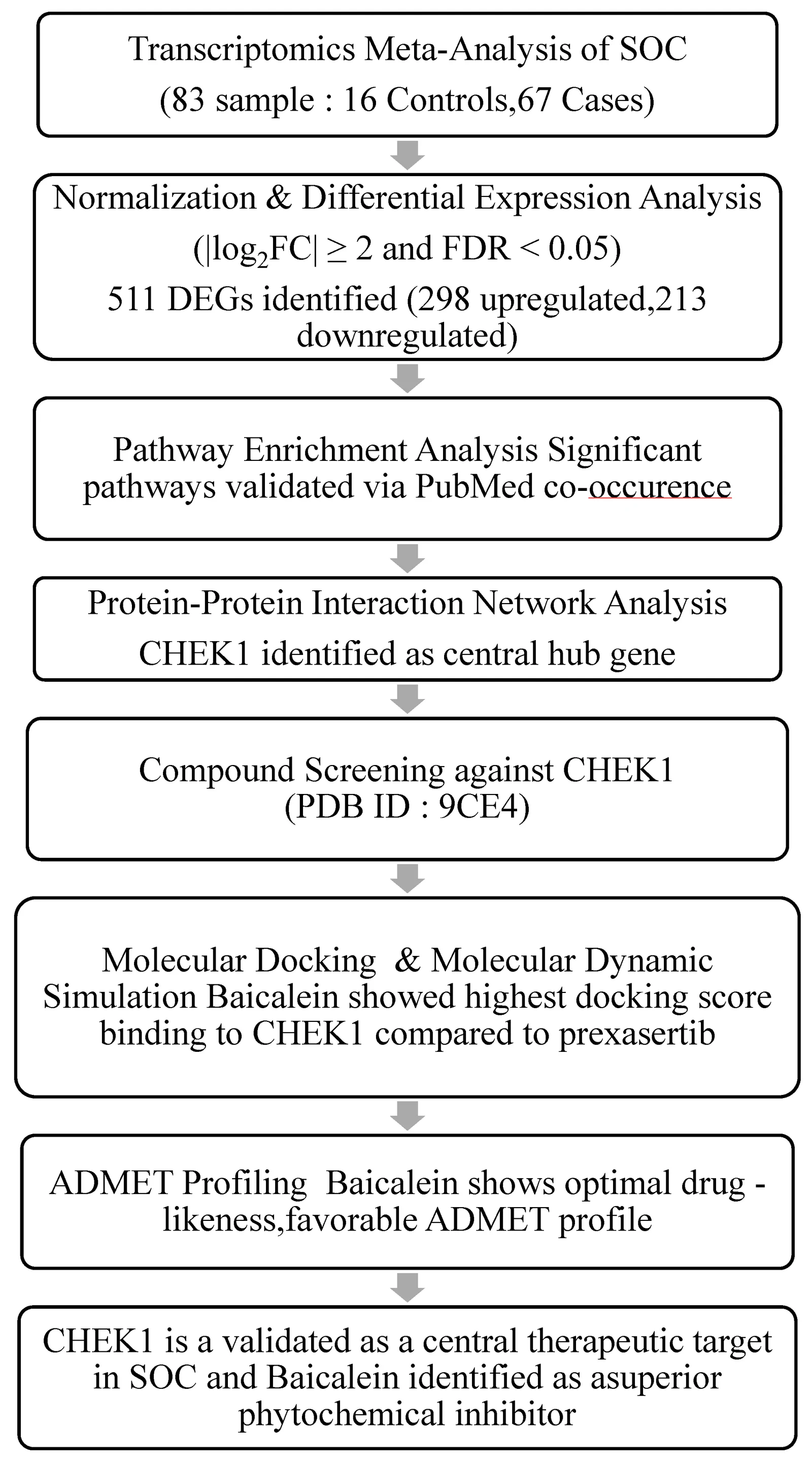
Workflow of integrative bioinformatics and computational analysis for identification of potential CHEK1 inhibitors in serous ovarian cancer.

This study employed a seven stage integrative in silico pipeline: (1) GEO dataset retrieval and its processing; (2) meta-analysis and differential gene expression (DEG) identification; (3) Reactome and KEGG pathway enrichment analysis; (4) PubMed literature-based pathway validation; (5) PPI network construction and hub gene identification; (6) molecular docking of phytochemical against CHEK1; and (7) molecular dynamic simulation and ADMET profiling (Figure 11).

### 4.2. GEO Dataset Retrieval and Preprocessing

Three microarray gene expression data sets related to high grade series ovarian cancer were retrieved from the Gene Expression Omnibus database maintained by NCBI, using the search strategy: “serous ovarian cancer” or “high grade serous ovarian carcinoma” and “microarray”. Datasets GSE27651 (Wong et al., United States), GSE36668 (Elgaaen et al., Norway), and GSE54388 (Yeung et al., Hong Kong) were selected based on platform consistency (all on Affymetrix Human Genome U133 Plus 2.0 Array, GPL570), sample availability, and biological relevance. The combined dataset comprised 83 samples (16 controls, 67 SOC cases). The raw data were pre-processed and the batch effect was normalized using the ImaGEO meta-analysis platform [43].

### 4.3. Meta-Analysis and DEG Identification

A meta-analysis was conducted using ImaGEO, applying stringent thresholds of absolute log_2_ fold change (|log_2_FC| ≥ 2) and a Benjamini–Hochberg false discovery rate (FDR)-adjusted *p*-value < 0.05. These criteria ensured that the identification of biologically significant and statistically robust DEGs consistently altered all three independent datasets.

### 4.4. Pathway Enrichment Analysis

DEGs were subjected to functional enrichment analysis using Enrichr [44]. The Reactome pathway database [45] was selected as the primary enrichment resource due to its comprehensive, manually curated, human-specific biological pathway annotations. Supplementary enrichment analysis used the KEGG 2026 database. Pathways with an adjusted *p*-value < 0.05 were considered significantly enriched.

### 4.5. Literature-Based Pathway Validation

To independently validate the biological relevance of enriched pathways, PubMed co-occurrence mining [46] was performed using a custom python script employing the Biopython Entrez module. For each enriched Reactome pathway, the frequency of co-occurrence with SOC-related search terms in the PubMed abstract were quantified by Fisher’s Exact Test, which assessed whether co-occurrence exceeded the background expectation with a Benjamini–Hochberg FDR correction applied to control the false discovery rate across multiple hypothesis tests. Pathways with adjusted *p*-value < 0.05 were considered independently validated.

### 4.6. PPI Network Analysis and Hub Gene Identification

Validated pathway genes were submitted to STRINGv12.0 [47] for PPI network construction using experimental data, co-expression, and curated database annotations. The network was imported into Cytoscapev3.10.2 [48] for visualization. Hub genes were identified using cytoHubba plugin (degree centrality) and the MCODE plugin for cluster detection.

### 4.7. Bioinformatics Validation via GEPIA2 (External Cohort)

To provide external validation beyond PPI centrality, CHEK1 expression and prognostic significance were assessed independently using the GEPIA2 web platform (http://gepia2.cancer-pku.cn, accessed on 15 January 2026) [15,16], which integrates TCGA tumor and GTEx normal tissue data with standardized preprocessing. Differential expression was evaluated for the ovarian serous cystadenocarcinoma (OV) cohort (tumor: n = 426; matched normal: n = 88) by using a one-way ANOVA-based method with |log_2_FC| > 1.0 and *p* < 0.01 as cutoffs. Statistical significance in the box-plot output is indicated by an asterisk (*). Overall survival (OS) analysis was performed using the Kaplan–Meier method with the median expression as the stratification threshold across 1435 OV patients [36]. Hazard ratios (HR) and 95% confidence intervals were derived from a Cox proportional hazards model; significance was assessed by the log-rank test. A significance-versus-cutoff analysis was performed across the interquartile range of expression values to confirm the threshold robustness.

### 4.8. Target Protein Preparation and Phytochemical Library

The CHEK1 crystal structure was retrieved from the RCSB Protein Data Bank [49] (PDB ID: 9CE4). Protein preparation included the removal of water molecules, addition of hydrogen atoms, and energy minimization. Forty anticancer phytochemicals were compiled from the published literature, screened for drug-likeness using Lipinski’s Rule of Five via SwissADMEv2.0 [50], and prepared for docking in PDBQT format using AutoDockToolsv1.5.7. Toxicity class was predicted using Pro Tox-II v5.0 [51].

### 4.9. Molecular Docking

The three-dimensional crystal structure of the CHEK1 protein (PDB ID:9CE4) was retrieved from the Protein Data Bank. Protein preparation involved the removal of water molecules, addition of missing hydrogen atoms, and energy minimization to stabilize the structure. Phytochemical compounds were selected based on evidence from the literature and prepared for docking by converting them into appropriate file formats. Molecular docking was performed using AutoDock Vina [20], centered at (4.830 × −4.588 × 17.072). A grid box centered on the CHEK1 ATP-binding pocket (exhaustiveness = 8). The binding interactions were characterized using UCSF Chimera v1.18 and Maestro Viewer v13.1.

### 4.10. Molecular Dynamics Simulation

The Desmond 2025-1 from Schrödinger, LLC, was used to run MD simulations on dock complexes CHEK 1-Baicalein and CHEK1–Prexasertib. The OPLS-2005 force [52,53,54] and explicit solvent model with the SPC water molecules were used in this system [55] in a period boundary solvation box of 10 Å × 10 Å × 10 Å dimensions. Na+ ions were added to neutralize the charge of 0.15 M, and NaCl solutions were added to the system to simulate the physiological environment. Initially, the system was equilibrated using an NVT ensemble for 10 ns to retrain over the protein–ligand complexes. Following the previous step, a short run of equilibration and minimization was carried out using an NPT ensemble for 12 ns. The NPT ensemble was set up using the Nose–Hoover chain coupling scheme [56] with varying temperatures, a relaxation time of 1.0 ps, and pressure of 1 bar maintained in all the simulations. A time step of 2 fs was used. The Martyna–Tuckerman–Klein chain coupling scheme [57] barostat method was used for pressure control with a relaxation time of 2 ps. The particle mesh Ewald method [58] was used for calculating long-range electrostatic interactions, and the radius for the coulomb interactions was fixed at 9 Å. The final production run was carried out for 100 ns. The root mean square deviation (RMSD), the radius of gyration (Rg), root mean square fluctuation (RMSF), and several hydrogens (H-bonds) are calculated to monitor the stability of the MD simulations [59].

#### 4.10.1. Binding Free Energy Analysis

The molecular mechanics combined with the generalized Born surface area (MM-GBSA) approach [60] was used to compute the binding free energies of the ligand–protein complexes. The Prime MM-GBSA binding free energy was calculated using the Pythonv3.10.12 script thermal mmgbsa.py in the simulation trajectory for the last 50 frames with a one-step sampling size. The binding free energy of Prime MM-GBSA (kcal/mol) was estimated using the principle of additivity, in which individual energy modules such as columbic, covalent, hydrogen bond, van der Waals, self-contact, lipophilic, solvation of protein and ligand were collectively added. The equation used to calculate Δ*G_bind_* is the following:

$${\Delta G}_{bind}={\Delta G}_{MM}+{\Delta G}_{Solv}-{\Delta G}_{SA}$$

where Δ*G_bind_* designates the binding free energy, Δ*G_MM_* designates the difference between the free energies of ligand–protein complexes and the total energies of the protein and ligand in isolated form, Δ*G_Solv_* designates the difference in the GSA solvation energies of the ligand–receptor complex and the sum of the solvation energies of the receptor and the ligand in the unbound state, and Δ*G_SA_* designates the difference in the surface area energies for the protein and the ligand [61].

CHEK 1–Baicalein and CHEK1–Prexasertib complexes were subjected to 100 ns MD simulation using Maestro–Desmond (Desmond v6.4, Schrodinger Inc., New York, NY, USA) [52] with the OPLS-2005 force field. Systems were solvated in orthorhombic boxes (10 A buffer, SPC water model), neutralized with 0.15 M Nacl, and protonated at pH 7.0. Simulations were conducted under NPT conditions at 300 K and 1 atm [62]. Trajectories were analyzed for protein backbone RMSD, residue-level RMSF, secondary structure element (SSE) content, ligand RMSD, radius of gyration (rGyr), protein–ligand contact fractions and ligand torsional profiles using the simulation Interaction Diagram tool in Maestro v13.1.

#### 4.10.2. RMSD Statistical Analysis and Comparative Stability Assessment

Cα RMSD trajectories were extracted for the production phase (20–100 ns) of both complexes. To reduce temporal autocorrelation, the production phase was divided into four equal non-overlapping blocks (20–40 ns, 40–60 ns, 60–80 ns, 80–100 ns) and block-mean RMSD values were calculated. Normality within each block was assessed by the Shapiro–Wilk test (α = 0.05); because several blocks deviated from normality, a two-sided Mann–Whitney U test [23] was applied to evaluate whether RMSD values of the Baicalein complex were significantly lower than those of the Prexasertib complex within each block (significance threshold *p* < 0.05). Descriptive statistics (mean ± SD) were calculated for both complexes over the full production phase.

To verify the internal consistency of the analytical pipeline, a replicate trajectory (RSMD2) was generated by duplicating the Baicalein production-phase trajectory and subjecting it to identical preprocessing and statistical treatment. Equivalence between RSMD1 and RSMD2 was assessed using the Two One-Sided Tests (TOST) procedure [24] with an equivalence margin of ±0.2 Å, confirming that the statistical workflow is reproducible and free of preprocessing artifacts.

#### 4.10.3. Dynamic Cross-Correlation Matrix(DCCM)

DCCM analysis was performed on the Cα coordinate time series of the Baicalein–CHEK1 complex using the correlation-based framework of Ichiye and Karplus [25]. Cross-correlation coefficients were calculated as C*ij* = <Δr*i*Δr*j*>/(⟨Δr*i*^2^⟩⟨Δr*j*^2^⟩)^0.5^, where Δr*i* denotes the displacement vector of residue i from its mean position. Positive values (blue) indicate concerted motion; negative values (red) indicate anti-correlated motion.

#### 4.10.4. Principal Component Analysis

PCA was applied to the Cα positional covariance matrix of the equilibrated trajectory using the Geo_measures PyMOLv3.1.6.1 plugin with g_sham as the computational backend [27]. The trajectory was superimposed by least-squares fitting prior to diagonalization. Projections were computed for PC1 vs. PC2, PC2 vs. PC3, and PC9 vs. PC10 pairs; frames were colored by simulation time to reveal temporal drift patterns. A scree plot was constructed for the first 20 principal components to visualize the cumulative variance profile.

#### 4.10.5. Free Energy Landscape Construction

The FEL was constructed by projecting the production-phase trajectory onto the RMSD and radius of gyration (Rg) and computing the 2D population histogram with a bin width of 0.25 Å in both dimensions. The Gibbs free energy at each bin was derived from the Boltzmann relation ΔG = −k*B*T ln(P/P*max*), where T = 300 K [28]. Uninhabited bins were assigned ΔG = 12 kJ/mol as a practical upper boundary. The surface was rendered as both a 2D contour map and a 3D surface plot.

#### 4.10.6. Interaction Persistence Analysis

Residue-wise interaction fractions were computed from the 100 ns trajectory using the Schrödinger Simulation Interaction Diagram tool [21]. Contacts were classified as hydrogen bonds, hydrophobic contacts, ionic interactions, and water bridges, using standard geometric criteria (H-bond: donor–acceptor distance ≤ 3.5 Å, angle ≥ 120°; hydrophobic: carbon–carbon distance ≤ 4.5 Å).

### 4.11. ADMET Profiling

Pharmacokinetic and drug-likeness properties were evaluated using SwissADME [50]. The assessed parameters included Lipinski’s Rule of Five compliance, gastrointestinal absorption, BBB permeability, P-glycoprotein substrate likelihood, CYP450 inhibition, solubility, and bioavailability score. Toxicity profiling used ProTox-II [51] for AMES mutagenicity, hepatotoxicity, and LD50 class prediction.

## 5. Conclusions

This study establishes CHEK1 as a computationally validated central therapeutic target in serous ovarian cancer through convergent transcriptomic, pathway, and network biology evidence, and identifies Baicalein as a superior natural inhibitor outperforming the Phase II clinical compound Prexasertib across all computational metrics evaluated. The key conclusions are that 511 high-confidence DEGs (298 upregulated, 213 downregulated) were identified from a meta-analysis of three GEO datasets (GSE27651, GSE36668, GSE54388). Eight Reactome and KEGG pathways were significantly enriched and independently validated by PubMed mining; DNA repair (OR = 31.89); and apoptosis (OR = 10.58), which shows the strongest associations. CHEK1 was identified as the central PPI hub gene interacting with nine key mitotic and DNA repair partners. Baicalein showed the highest docking score (−9.334 kcal/mol) among 40 phytochemicals, exceeding Prexasertib (−7.2 kcal/mol) by 2.134 kcal/mol. ADMET profiling confirmed Baicalein as drug-like: zero Lipinski violations, high GI absorption, no mutagenicity, and no hepatotoxicity (Toxicity Class 5). The 100 ns MD simulation confirmed superior CHEK1–Baicalein dynamic stability: ligand RMSD 0.2–0.6 A vs. 1.0–2.4 A (Prexasertib); SSE 42.30% vs. 38.62%; and 92% docking-MD concordance. Water-mediated interactions (LYS38 58%; GLU55 22%) identified via MD represent a novel binding stabilization mechanism.

These computational pharmacokinetic properties, while requiring experimental confirmation, support Baicalein as a drug-like candidate scaffold for further development. It must be acknowledged that docking scores represent approximate binding free energies and cannot establish biochemical potency; experimental IC_50_ determination and cellular target engagement assays are required before superiority over Prexasertib can be claimed.

A 100 ns all-atom molecular dynamics simulation of the CHEK1–Baicalein complex provided comprehensive characterization of dynamic stability and binding persistence. The Baicalein complex maintained a ligand RMSD of 0.2–0.6 Å and secondary structure element (SSE) retention of 42.30%, compared with 1.0–2.4 Å and 38.62% for the Prexasertib complex, with 92% concordance between the docking pose and the dominant MD-derived binding geometry. Block-wise two-sided Mann–Whitney U testing across four independent 20 ns production-phase windows confirmed that the Baicalein complex maintained significantly lower Cα RMSD values in all blocks (*p*-values 2.34 × 10^−55^ to 2.42 × 10^−67^; all *p* < 0.001), with complete stochastic separation (U = 0) in the 40–60 ns window. Pipeline reproducibility was confirmed by TOST equivalence testing of the original and duplicate Baicalein trajectories (t = ±2.325, *p* = 0.017, equivalence margin ± 0.2 Å). Dynamic cross-correlation analysis revealed a coherent allosteric communication network; principal component analysis demonstrated that three eigenvectors captured > 94% of the total positional variance (PC1: 66.08%); and free energy landscape profiling identified a single dominant low-energy basin (ΔG ≈ 0 kJ/mol) at RMSD 1.5–3.5 Å and Rg 19.5–20.1 Å, confirming thermodynamic convergence. Interaction persistence analysis identified near-continuous hydrogen-bonding contacts at GLU85 and ALA87, augmented by water-mediated stabilization at LYS38 (58% occupancy) and GLU55 (22% occupancy)—a novel hydration-dependent binding stabilization mechanism not observed in the Prexasertib complex.

Collectively, the convergent computational evidence from transcriptomic meta-analysis, external TCGA survival validation, validated molecular docking, ADMET profiling, and multi-parameter MD analysis identifies Baicalein as a lead-stage natural inhibitor scaffold for CHEK1-targeted therapy in SOC. All findings are explicitly framed as in silico evidence that generates mechanistically grounded hypotheses for experimental testing; no claims of clinical superiority over Prexasertib are made on the basis of computational data alone. The scientific foundation provided by this study supports a structured translational research program comprising: (i) in vitro validation of CHEK1 enzyme inhibition and antiproliferative activity in SKOV-3 and OVCAR-3 cell lines by MTT assay, apoptosis quantification, and target engagement assays; (ii) in vivo murine xenograft efficacy and pharmacokinetic assessment; (iii) structure–activity relationship optimization of the Baicalein scaffold to improve potency and selectivity; and (iv) development of nanoparticle-based delivery systems to enhance aqueous solubility, bioavailability, and tumor targeting. Successful progression through these stages would establish Baicalein as a clinically viable natural-product-derived CHEK1 inhibitor for serous ovarian cancer.

## Figures and Tables

**Table 1:** Literature-based validation of enriched Reactome pathways in serous ovarian cancer (PubMed co-occurrence mining; Fisher’s Exact Test; Benjamini–Hochberg FDR correction).

Pathway	Pathway Papers	Overlap	Odds Ratio	*p*-Value	Adj. *p*-Value
Apoptosis	533,080	47	10.58	2.00 × 10^−30^	1.81 × 10^−27^
DNA Repair	59,334	17	31.89	5.72 × 10^−20^	2.59 × 10^−17^
Cell Cycle	212,183	24	12.79	1.97 × 10^−18^	5.96 × 10^−16^
Disease	4,569,420	105	3.00	2.95 × 10^−18^	6.67 × 10^−16^
Autophagy	97,011	10	11.24	4.33 × 10^−8^	7.83 × 10^−6^
DNA Replication	42,221	6	15.35	3.73 × 10^−6^	5.62 × 10^−4^
Programmed Cell Death	42,574	5	12.65	5.99 × 10^−5^	7.75 × 10^−3^
Metabolism	919,289	22	2.64	7.86 × 10^−5^	8.89 × 10^−3^

**Table 2:** Top 10 phytochemicals ranked by AutoDock Vina binding affinity against CHEK1 (PDB: 9CE4). Prexasertib, a synthetic CHEK1 inhibitor, is included as a reference compound and is not ranked among the phytochemicals.

S. No	Phytochemical	Class	Source Plant	Binding Score (kcal/mol)
1	Baicalein	Flavone	*Scutellaria baicalensis*	−9.334
2	Tanshinone IIA	Diterpene	*Salviamiltio rhiza*	−9.273
3	Emodin	Anthraquinone	*Polygonum* spp.	−8.590
4	Gambogic acid	Xanthone	*Garcinia hanburyi*	−8.543
5	Polydatin	Stilbenoid	*Polygonum cuspidatum*	−8.505
6	Silymarin	Flavonolignan	*Silybum marianum*	−8.471
7	Diosgenin	Steroid	*Dioscorea* spp.	−8.442
8	Rhein	Anthraquinone	*Rheum officinale*	−8.389
9	EGCG	Polyphenol	*Camellia sinensis*	−8.373
10	Quercetin	Flavonoid	*Capparis spinosa*	−8.341
Ref	Prexasertib	Small molecule inhibitor	Synthetic (CHEK1 inhibitor)	−7.2

**Table 3:** ADMET and pharmacokinetic properties of top-ranked phytochemicals against CHEK1.

Compound	Toxicity Class	LD_50_ (mg/kg)	Mutagenicity (Ames)	Carcinogenicity
Baicalein	Class 5	2887	Non-mutagenic	Non-carcinogenic
Tanshinone IIA	Class 3	1000	Weakly mutagenic	Non-carcinogenic
Emodin	Class 3	114	Mutagenic	Carcinogenic
Gambogic acid	Class 4	500	Non-mutagenic	Non-carcinogenic
Polydatin	Class 4	2500	Non-mutagenic	Non-carcinogenic
Silymarin	Class 5	10,000	Non-mutagenic	Non-carcinogenic
Diosgenin	Class 4	3200	Non-mutagenic	Inconclusive
Rhein	Class 3	320	Mutagenic	Carcinogenic
EGCG	Class 4	3981	Non-mutagenic	Non-carcinogenic
Quercetin	Class 4	159	Weakly mutagenic	Non-carcinogenic

**Table 4:** Block-wise Cα RMSD statistics for Baicalein and Prexasertib–CHEK1 complexes (production phase 20–100 ns).

Time Block	Baicalein Mean RMSD (Å)	Prexasertib Mean RMSD (Å)
20–40 ns	2.50 ± 0.24	3.24 ± 0.38
40–60 ns	2.37 ± 0.11	3.52 ± 0.24
60–80 ns	2.69 ± 0.24	3.46 ± 0.20
80–100 ns	2.61 ± 0.22	3.16 ± 0.26
Full phase	2.61 ± 0.28	3.16 ± 0.41

**Table 5:** Comparative MD simulation summary: CHEK1–Baicalein vs. CHEK1–Prexasertib (Desmond, NPT, 300 K, 100 ns).

Parameter	CHEK1–Baicalein	CHEK1–Prexasertib
Docking score (kcal/mol)	−9.334	−7.2
Simulation time (ns)	100.102	100.102
Protein RMSD (A)	1.0–2.0 (stable)	1.2–3.2 (higher)
Ligand RMSD (A)	0.2–0.6 (very stable)	1.0–2.4 (moderate)
Total SSE (%)	42.30%	38.62%
Alpha-helix content (%)	25.08%	21.60%
Beta-strand content (%)	17.22%	17.02%
Strongest contact	ALA36: ~80% hydrophobic	GLY16: ~82% H-bond
Ligand rGyr (A)	3.90–4.02 (compact)	4.50–5.25 (extended)
Water-mediated interactions	LYS38 (58%), GLU55 (22%)	None significant
Docking-MD concordance	92% (11/12 residues)	~93% with reorganization

## Data Availability

The data presented in this study are available in the Gene Expression Omnibus (GEO) database (https://www.ncbi.nlm.nih.gov/geo/) (accessed on 15 July 2025) under accession numbers GSE27651, GSE36668, and GSE54388, and the RCSB Protein Data Bank (https://www.rcsb.org/) (accessed on 25 August 2025) under PDB ID: 9CE4.
